# The risk of atrial fibrillation in patients with gout: a nationwide population-based study

**DOI:** 10.1038/srep32220

**Published:** 2016-09-07

**Authors:** Yu-Jui Kuo, Tzu-Hsien Tsai, Hui-Ping Chang, Sarah Chua, Sheng-Ying Chung, Cheng-Hsu Yang, Cheng-Jei Lin, Chiung-Jen Wu, Chi-Ling Hang

**Affiliations:** 1 Department of Traditional Chinese Medicine, Tainan Municipal Hospital, Tainan, Taiwan; 2 Division of Cardiology, Department of Internal Medicine, Kaohsiung Chang Gung Memorial Hospital and Chang Gung University College of Medicine, Kaohsiung, Taiwan; 3 Department of Chinese Medicine, Sin-Lau Hospital, Tainan, Taiwan.

## Abstract

Many studies have found that systemic inflammation plays an important role in the pathogenesis of atrial fibrillation (AF). Gout is a chronic systemic inflammatory disorder, but little evidence exists regarding whether the risk of AF is increased in patients with gout. The National Health Insurance Research Database in Taiwan was used in this study, and gout was defined as the occurrence of at least one episode of an acute gout attack requiring medical treatment. A total of 63264 gout and 63264 age- and gender-matched patients were included as the study population. The Cox model was used to evaluate the risk of AF in patients with gout. Patients with gout experienced a greater frequency of co-morbidities compared to patients without gout. The cumulative incidences of AF were 4.61% and 3.04% in patients with and without gout, respectively (log-rank test, P < 0.001). After adjusting for co-morbidities and prescription medication use, gout was found to be associated with AF [hazard ratio (HR), 1.38]. Moreover, the HR for AF decreased with increasing age in our study. Gout was found to be associated with an increased risk of developing AF after adjusting for potential confounders.

The link between inflammation and cardiovascular diseases such as atrial fibrillation (AF), coronary artery disease (CAD) and myocardial infarction has been established^1^,^2^. Gout, which is a chronic and systemic inflammatory arthritis caused by hyperuricemia, has been found to be associated with metabolic syndrome^3^,^4^. The presence of metabolic syndrome (MS) is also associated with a higher prevalence of cardiovascular disease^5^,^6^,^7^,^8^ However, the prevalence of gout has been increasing worldwide^9^,^10^,^11^,^12^,^13^. More importantly, gout has been shown to be associated with cardiovascular comorbidity, and it is also an independent risk factor for cardiovascular death^9^,^10^,^11^,^12^,^13^. AF is the most common cardiac arrhythmia and is also an independent risk factor for stroke^14^,^15^. The incidence of AF has been predicted to increase over the next few decades^14^,^15^. Many studies have suggested that systemic inflammation plays an important role in the initiation, maintenance and recurrence of AF^16^,^17^,^18^. In addition, the incidence of atrial fibrillation is known to increase with several risk factors such as diabetes mellitus, hypertension and obesity^19^,^20^,^21^. Interestingly, all of these risk factors are components of MS^19^,^20^,^21^. Moreover, a potential link between gout and MS has been repeatedly suggested^3^,^4^. Based on these findings, we assume that there may be a link between AF and gout, although to the best our knowledge, there are few studies exploring the relationship between AF and gout. In this study, therefore, we estimated the incidence of AF among individuals with gout and determined the magnitude of the association between gout and AF in the population cohort.

## Results

### Cohort Selection

**Figure 1:**
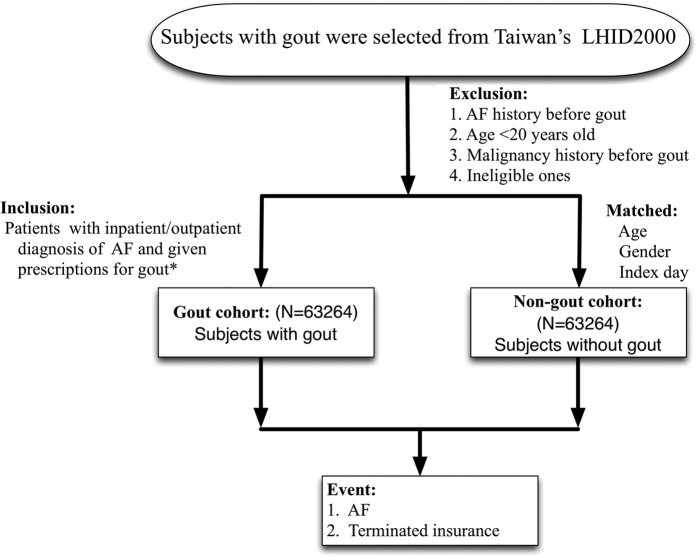
Cohort selection flow chart. The final study cohort included 63264 patients with gout and 63264 patients without gout matched by age, gender and index day. AF: atrial fibrillation; LHID: Longitudinal Health Insurance Database *Prescriptions include: colchicine, benzbromarone, allopurinol, probenecid, or sulfinpyrazone.

The patients were selected from the NHIRD, which is a cohort dataset that contains all medical claims data for 1,000,000 individuals who were randomly sampled from the 23 million administered insurants under the NHI program. The database has a large sample size and provided us with an excellent opportunity to study the risk of AF in patients with gout. The selection criteria and process for this study cohort are displayed in Fig. 1. There were initially 63264 patients with at least one gout attack diagnosed after a 365-day enrollment period. After applying the inclusion and exclusion criteria and comparing the gout and the non-gout groups, 63264 gout patients were matched by age, sex, and index date to non-gout patients in a 1:1 ratio and were included in the study. Our final study cohort included 63264 gout and 63264 non-gout control patients.

### Patient Characteristics

After applying the inclusion and exclusion criteria, the study included 63264 gout subjects (47070 male and 16194 female) and 63264 non-gout subjects (47070 male and 16194 female). The baseline characteristics of the age-, sex- and index date-matched groups were compared between the gout and non-gout groups (Table 1). The mean age of patients in the study cohort was 51.29 years, and men composed 74.4% of both groups. Patients with gout had a higher frequency of hypertension, diabetes mellitus (DM), chronic kidney disease (CKD), hyperlipidemia, chronic obstructive pulmonary disease (COPD), coronary artery disease (CAD), history of cardiac or pulmonary surgery, alcoholic intoxication, sleep apnea, congestive heart failure (CHF), rheumatoid arthritis (RA), and systemic lupus erythematosus (SLE) compared to the non-gout group. The mean follow-up duration was 6.1 ± 2.1 years, and there were 2917 patients (4.61%) who experienced AF with an annual AF rate of approximately 4.9%. Interestingly, the overall incidence of atrial fibrillation was approximately 50% higher in the patients with gout arthritis than in the general population (age, sex and index-day matched).

### The impact of co-morbidity, gender and age in risk of AF in gout patients

The adjusted HR for the incidence of AF with and without co-morbidity within the study cohorts is shown in Table 2. Interestingly, the adjusted HR for the incidence of AF was significant both with and without COPD comorbidity. However, the adjusted HR for the incidence of AF was not significantly increased in patients with comorbid DM, CKD, history of cardiac or pulmonary surgery, alcoholic intoxication, sleep apnea, RA, SLE or Sjögren’s syndrome (SS). A separate analysis for gender and age was performed to further investigate the impact of gout on the occurrence of AF in Table 3. A difference in risk for AF by sex was not apparent, although men had a slightly higher risk of AF than women (HR, 1.41; 95% CI 1.29 to 1.55 among men vs HR, 1.29; 95% CI 1.12 to 1.48 among women), which indicated no sex-specific effect modification. However, younger subjects with gout (age < 65) demonstrated a higher risk of occurrence of AF than elderly subjects (age ≥ 65) (adjusted HR 1.62; 95% CI 1.42 to 1.82 vs. adjusted HR 1.25; CI 1.14 to 1.38).

### Gout and the risk of AF

**Figure 2:**
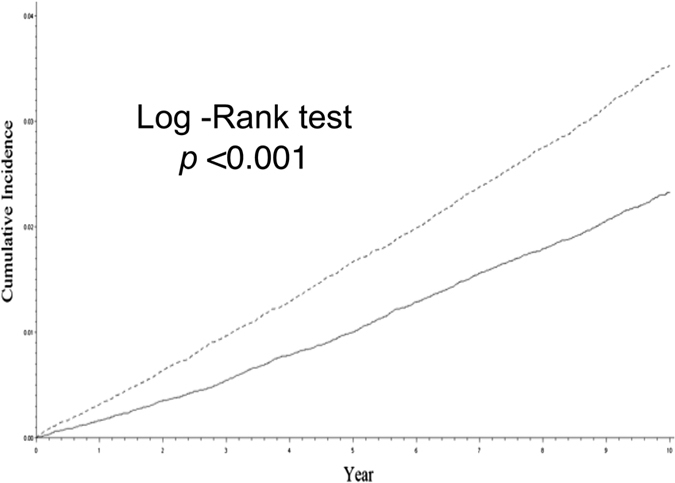
Cumulative incidence of atrial fibrillation in patients with gout and age-, gender- and index day matched cohorts. A Kaplan–Meier survival analysis showing that gout patients are associated with a higher incidence of AF compared to those without gout (16.8% versus 13.9%, p < 0.002). AF = atrial fibrillation.

A Kaplan-Meier curve was used to compare the cumulative incidences of AF in the age-, sex- and index date-matched gout and non-gout groups, and it showed a higher cumulative incidence of AF in patients with gout. (Fig. 2) The multivariable Cox proportional model was used to calculate adjusted HRs for the incidence of AF and is shown in Table 4. The traditional risk factors such as HTN, CAD and CHF were all independent predictors for AF. Interestingly, after adjustment for the extended range of covariates, gout remained an independent risk factor for AF.

## Discussion

### Main findings

In this study, we determined the risk of AF in patients with and without gout in a nationwide cohort enrolling 126528 subjects. The main findings were as follows: first, this study showed that patients with gout had more comorbid diseases and a higher frequency of prescription drug use than patients without gout; second, gout was associated with an increased risk of developing AF after adjusting for potential confounders including age, gender and co-morbidities; and third, another important finding of this present study was the greater impact of gout as a risk factor for the occurrence of AF, especially in young patients.

### Potential mechanisms of risk for AF in patients with Gout

The reasons for the increased risk of AF in patients with gout remain unclear. Accumulating evidence suggests that markers of systemic inflammation such as C-reactive protein, interleukin-6 and soluble adhesion molecules appear to play a role in the development and maintenance of AF^1^,^22^. Low grade systemic inflammation is associated with gout, and markers of systemic inflammation are often elevated during the both the acute attack and the chronic phase. Therefore, these findings suggest that systemic inflammation might promote the onset of AF in patients with gout. Evidence has shown that the link between AF and gout may be derived from their shared metabolic factors^3^,^4^,^19^,^20^,^21^. These factors, which include obesity, hypertension and diabetes mellitus, may cause greater levels of oxidative stress, chronic inflammation and endothelial dysfunction^20^,^21^,^23^. These factors increase left atrial size, which is an important determinant of AF and a factor that increases the risk of new-onset AF^20^,^21^,^23^. Moreover, hyperuricemia has been proven to be a risk factor for the development of AF in several cohort studies^24^,^25^. An increase in arterial stiffness and left atrial remodeling may be the major factors contributing to the occurrence of AF^26^, and both of these factors are also found in patients with gout^27^,^28^. Moreover, left atrial remodeling in patients with gout is more than hyperuricemia^27^. Some reports have also found that the use of non-steroidal anti-inflammatory drugs is a risk factor for AF^29^,^30^. This finding is another potential factor partially explaining why gout is a risk factor for AF.

### The impact of gender and age on the occurrence of AF in patients with Gout

The incidence of atrial fibrillation has not been investigated previously in a large gout population, and our study is the first to examine the occurrence of AF in gout patients in the nationwide registry population. Previous studies have shown that gout is more likely to be associated with cardiovascular diseases in females than males^9^,^31^,^32^. The gender difference in uric acid metabolism resulting in different serum uric acid levels may explain the phenomenon of stronger risk of MI associated with gout among women compared to men^9^,^32^. However, we did not observe a significant gender difference in gout-related AF risk. Racial differences^13^, heterogeneity of study design and choice of potential explanatory covariates may be some of the possible reasons explaining the divergent results between the present findings and previous studies^9^,^31^,^32^. Another interesting finding from the present study was that younger patients with gout had a higher incidence of AF than older patients with gout after adjusting for confounding factors. According to the results of the subgroup analyses in the present study, the hazard ratio for gout predicting AF was attenuated in patients who were older or who had any comorbidities. This finding was in accordance with results from previous studies showing that hyperuricemia- and rheumatoid arthritis-related AF risk decreased after adjusting for the occurrence of comorbidities^33^,^34^. There was a remarkable increase in the relative risks in patients younger than 65 years old with gout, which was also indicated by the high proportion of risk that was attributable to gout in young patients compared with older patients. These findings indicate that gout may represent a marker of cardiovascular risk^35^.

### Clinical implications

The findings of the present study have some implications for clinical practice. First, the management of gout in primary care remains suboptimal, and even a small increase in AF risk can lead to a substantial increase in new cerebral or vascular events in the future^36^. Because no evidence relating to the risk of atrial fibrillation in patients with gout has been available, it has often been overlooked. In addition, there is evidence that cardiovascular disease in patients with gout is often unrecognized and undertreated in real-world practice, despite recent guideline recommendations^37^,^38^. Therefore, aggressive management of patients with gout is strongly recommended in clinical practice. Second, traditional risk factors for AF, such as patient age, hypertension, CKD and DM are well known^39^,^40^,^41^. However, several novel risk factors have subsequently been recognized including obstructive sleep apnea, obesity and the metabolic syndrome^39^,^40^,^41^. Our results suggest that gout may be another novel risk factor for AF, especially in the young population. Screening for AF is not currently recommended in clinical practice, although it should be considered for patients with gout who exhibit cardiac arrhythmia-related symptoms, especially in younger patients. However, the results of our present study cannot be used to determine whether the aggressive treatment of gout can reduce the risk of occurrence of AF.

Previous studies have found that hyperhomocysteinemia and hyperuricemia are both risk factors of developing cardiovascular diseases^42^,^43^,^44^,^45^. However, treatment of an elevated serum homocysteine level does not reduce cardiovascular events^46^,^47^, and treatment of hyperuricemia does not reduce the incidence of heart failure^48^,^49^. Therefore, this important and interesting issue requires a randomized, placebo-controlled trial to determine the effect of treatment of hyperuricemia on the incidence of atrial fibrillation.

### Strengths and weaknesses

We note several weaknesses and strengths of this study. First, we examined a large nationwide registry cohort of gout patients in a population, which is representative of the commercially insured Taiwanese population. To the best of our knowledge, this is the first population-based study to investigate the risk of AF in patients with gout. Although the increased incidence of atrial fibrillation in patients with gout is a novel finding, this finding must be further tested in other cohorts. Second, we depended primarily on diagnosis codes to identify cases of gout and AF, which may potentially cause outcome misclassification, although the ICD codes for both gout and AF have been used and validated in previous studies^13^,^34^. In addition, the treatment and diagnosis codes for gout rather than the diagnosis code alone were used to minimize surveillance bias in our study. This methodology should reduce the possibility of misclassification in a claims-based study. Third, information regarding the type of AF, e.g., paroxysmal or persistent, is lacking, although no difference in the risk of embolic events has been found between paroxysmal and non-paroxysmal AF^25^,^36^. Fourth, no information was available in the nationwide registry database related to some potential confounding covariates such as serum uric acid level, personal smoking history and body mass index. However, we attempted to partially adjust for lifestyle factors by matching a history of pulmonary diseases, hyperlipidemia and ischemic heart disease between the groups with and without gout.

## Conclusion

Our findings showed that gout is significantly associated with an increased risk of developing AF after adjusting for potential confounders. Furthermore, our results suggest that gout may be a novel risk factor for AF, especially in young patients. Because systemic inflammation and hyperuricemia may play important roles in the occurrence of AF, future research is needed to investigate the role of aggressive gout management including urate-lowering therapy on the risk of AF in the future.

## Methods

### Data source

The National Health Insurance Research Database (NHIRD), which is one of the largest and most comprehensive population-based health databases in the world, covers all hospitalization and outpatient medical claims for approximately 99% of the over 23 million people in Taiwan. The National Health Research Institutes (NHRI) has established several data files and released the insured medical records as de-identified secondary data to the public for the purpose of research^50^. We requested the NHIRD from the institute, which covers claims data from 1998 to 2010. The accuracy and completeness of the NHIRD have been guaranteed by the NHRI Bureau of Taiwan and the Department of Health. This study was conducted in full compliance with relevant local and national ethical and regulatory guidelines and was approved by the Institutional Review Board of Chang Gung Memorial Hospital (IRB:104-7652B). This retrospective population-based cohort study used data from the Longitudinal Health Insurance Database 2000 (LHID 2000), which is a subset of the NHIRD. The LHID 2000 contains the complete original claims data of one million insured individuals who were randomly sampled from the NHIRD registry in 2000. Until the end of 2010, all sampled individuals were followed up for outcome identification using the International Classification of Disease, 9th Revision, and Clinical Modification (ICD-9-CM).

### Study subjects

Based on the LHID 2000, we included patients who were diagnosed with gout (ICD-9-CM 274.X) for the first time between January 1, 2000 and December 31, 2007, and labeled these patients as the “gout cohort.” Gout patients were defined by one of the following criteria: (1) outpatients who were diagnosed with gout and given prescriptions for colchicine, benzbromarone, allopurinol, probenecid, or sulfinpyrazone during the same visit; and (2) inpatients with gout who were given prescriptions for colchicine, benzbromarone, allopurinol, probenecid, or sulfinpyrazone. The first day of diagnosis was defined as the index day. Comorbidities were classified as those existing prior to the index day and included DM, HTN, CKD, COPD, CAD, sleep apnea, CHF, SLE, history of cardiac or pulmonary surgery, rheumatoid arthritis, scleroderma, and Sjögren’s disease. We excluded patients with a diagnosis of malignancy or who had received chemotherapy in the 365-day period prior to the index date. Non-gout subjects were selected from the same dataset. Subjects were then matched one by one by age, gender, and index day. Enrolled subjects with a history of atrial fibrillation before the index day were excluded. The index date for the non-gout patients began at the first receipt of any prescription drug after at least 2 physician visits. Non-gout patients were not allowed to use allopurinol, sulfinpyrazone, colchicine, or probenecid in the 365-day period prior to or on the index date. The non-gout group was then matched to the gout group by age, sex and index date in a 1:1 ratio. The end of the follow-up period for the analyses was marked on the day of atrial fibrillation diagnosis, terminated enrollment from the NHI, death, or until the end of this study. To ensure that only newly diagnosed AF cases were detected for the endpoint, we excluded patients with a diagnosis of AF in the 365-day period prior to the index date of gout from both cohorts. Follow-up data were available for a minimum of five years for all subjects.

### Statistical analysis

The data are presented as the mean and standard deviation (SD) for normally distributed continuous variables and as proportions for categorical variables. Differences among groups were evaluated using the *t*-test for continuous variables and the Chi-square test for categorical variables. The cumulative incidence rates of atrial fibrillation were measured for both cohorts. We used the stratified Cox proportional hazards regression model to determine the HRs of subsequent atrial fibrillation for gout patients compared with the matched cohort. Covariates used in the model included age and comorbidities. We also separated subjects in both of the cohorts into strata, first using gender, then again using age (age < 65, age > = 65). The results were analyzed using the Cox model to calculate specific-hazard ratios. The Kaplan-Meier method was used to determine the cumulative incidence of AF in both cohorts, and differences between the cohorts were tested using the log-rank test. The HR showed that the confidence interval (CI) was 95% and the *p*-value was two-sided. All *p*-values less than 0.05 were considered significant. All data management and calculations of HRs were performed using the Statistical Analysis System (SAS) software (version 9.4; SAS Institute, Cary, NC). This manuscript was written following STROBE guidelines for the reporting of observational studies.

## Additional Information

**How to cite this article**: Kuo, Y.-J. *et al*. The risk of atrial fibrillation in patients with gout: a nationwide population-based study. *Sci. Rep.* **6**, 32220; doi: 10.1038/srep32220 (2016).

## Figures and Tables

**Table 1:** Characteristics of the Study Subjects.

	Matched cohort(N = 63,264)	Gout cohort(N = 63,264)	*p*-value
Age (Mean ± SD)	51.29 ± 16.25	51.29 ± 16.25	0.9890&
Age group			0.9874$
<20	1420 (2.24)	1415 (2.24)	
20–49	15106 (23.88)	15094 (23.86)	
50–64	26188 (41.39)	26197 (41.41)	
65–79	18891 (29.86)	18935 (29.93)	
≧80	1647 (2.60)	1623 (2.57)	
Gender			1.0000$
Female	16194 (25.60)	16194 (25.60)	
Male	47070 (74.40)	47070 (74.40)	
Comorbidities			
DM	4867 (7.69)	8933 (14.12)	<0.0001$
CKD	446 (0.70)	1087 (1.72)	<0.0001$
HTN	11153 (17.63)	23412 (37.01)	<0.0001$
Hyperlipidemia	4719 (7.46)	17119 (27.06)	<0.0001$
COPD	1583 (2.50)	2199 (3.48)	<0.0001$
CAD	4779 (7.55)	8868 (14.02)	<0.0001$
History of cardiac or pulmonary surgery	28 (0.04)	48 (0.08)	0.0217$
Alcoholic intoxication	91 (0.14)	242 (0.38)	<0.0001$
Sleep apnea	82 (0.13)	150 (0.24)	<0.0001$
CHF	1365 (2.16)	2783 (4.40)	<0.0001$
Rheumatoid arthritis	253 (0.4)	856 (1.35)	<0.0001$
SLE	51 (0.08)	84 (0.13)	0.0045$
Scleroderma	6 (0.01)	12 (0.02)	0.1573$
Sjögren’s syndrome	135 (0.21)	161 (0.25)	0.1303$
Atrial fibrillation	1924 (3.04)	2917 (4.61)	<0.0001$

The statistical analyses were performed by t-test or Chi-square test. Symbols (&, $) indicate significance (at *p* < 0.05 level). &: T-test; $: Chi-squared test; DM: diabetes mellitus; CKD: chronic kidney disease; HTN: hypertension; CHF: congestive heart failure; COPD: chronic obstructive pulmonary disease; CAD: coronary artery disease; SLE: systemic lupus erythematosus.

**Table 2:** Prediction of occurrence of atrial fibrillation stratified by comorbidities

Gout vs. matched	With comorbidity		Without comorbidity	
	cHRs (95% C.I.)	*p*-value	aHRs (95% C.I.)	*p*-value
Diabetes Mellitus	1.04 (0.88–1.22)	0.6543	1.53(1.42–1.64)	<0.0001
CKD	1.11 (0.71–1.72)	0.6570	1.50 (1.41–1.60)	<0.0001
HTN	0.98 (0.89–1.07)	0.6348	1.31 (1.20–1.43)	<0.0001
COPD	2.80 (1.01–7.77)	0.0481	1.61 (1.49–1.73)	<0.0001
CAD	1.22 (0.93–1.61)	0.1601	1.59 (1.46–1.74)	<0.0001
History of cardiac or pulmonary surgery	1.33 (0.40–4.38)	0.6404	1.52 (1.42–1.62)	<0.0001
Alcoholic intoxication	1.26 (0.26–6.06)	0.7748	1.52 (1.43–1.62)	<0.0001
Sleep apnea	0.75 (0.17–3.37)	0.7102	1.52 (1.43–1.62)	<0.0001
CHF	0.93 (0.77–1.13)	0.4512	1.48 (1.39–1.59)	<0.0001
Rheumatoid arthritis	2.20 (0.87–5.57)	0.0949	1.51 (1.42–1.61)	<0.0001
SLE	0.97 (0.23–4.06)	0.9669	1.52 (1.43–1.62)	<0.0001
Scleroderma	—		1.52 (1.42–1.62)	<0.0001
Sjögren’s syndrome	0.91 (0.33–2.51)	0.8568	1.52 (1.43–1.62)	<0.0001

The statistical analysis was performed with the Cox proportional hazards regression model to indicate significance (at *p* < 0.05 level).

aHRs: adjusted hazard ratios; cHRs: crude hazard ratios; CI: confidence interval; other abbreviations as in Table 1.

**Table 3:** Prediction of occurrence of atrial fibrillation stratified by gender and age.

	Male		Female		<65		> = 65	
	cHRs (95% C.I.)	*p*-value	aHRs (95% C.I.)	*p*-value	cHRs (95% C.I.)	*p*-value	aHRs (95% C.I.)	*p*-value
Gout vs. matched	1.41 (1.29–1.55)	<0.0001	1.29 (1.12–1.48)	0.0005	1.62 (1.42–1.84)	<0.0001	1.25 (1.14–1.38)	<0.0001
Age	1.33 (1.06–1.65)	0.0121	1.08 (0.79–1.48)	0.6185	1.36 (1.00–1.84)	0.0477	1.17 (0.93–1.47)	0.1776
Comorbidities								
DM	0.98 (0.81–1.19)	0.8333	1.47 (1.16–1.85)	0.0013	1.37 (1.04–1.80)	0.0233	1.05 (0.88–1.25)	0.6184
CKD	1.77 (1.08–2.91)	0.0239	0.97 (0.53–1.78)	0.9271	3.01 (1.12–8.10)	0.0291	1.27 (0.82–1.95)	0.2849
HTN	1.21 (1.05–1.40)	0.0101	1.31 (1.06–1.62)	0.0114	1.35 (1.10–1.67)	0.0049	1.15 (0.99–1.33)	0.0659
COPD	1.01 (0.79–1.29)	0.9496	0.87 (0.56–1.35)	0.5330	1.08 (0.60–1.97)	0.794	0.98 (0.78–1.23)	0.8423
CAD	1.57 (1.32–1.87)	<0.0001	1.43 (1.13–1.79)	0.0026	1.59 (1.20–2.12)	0.0014	1.50 (1.28–1.77)	<0.0001
HCPS*	1.99 (0.37–10.69)	0.4203	—		—		2.13 (0.41–11.06)	0.3673
AI^	1.76 (0.50–6.19)	0.3823	—		0.90 (0.19–4.38)	0.8997	4.17 (0.44–39.45)	0.2132
Sleep apnea	1.45 (0.37–5.66)	0.5964	—		1.00 (0.14–7.46)	0.9986	1.23 (0.20–7.48)	0.8211
CHF	3.40 (2.48–4.68)	<0.0001	2.21 (1.58–3.10)	<0.0001	9.90 (4.13–23.75)	<0.0001	2.37 (1.85–3.02)	<0.0001
RA	0.84 (0.41–1.74)	0.6408	1.16 (0.57–2.36)	0.6745	1.39 (0.60–3.23)	0.4378	0.88 (0.46–1.68)	0.7016
SLE	0.73 (0.10–5.27)	0.7534	—		2.20 (0.19–25.66)	0.5299	1.60 (0.13–20.22)	0.7146
Scleroderma	—	—	—		0.46 (0.02–10.61)	0.6272		
SS&	1.00 (0.30–3.29)	0.9995	1.36 (0.36–5.18)	0.6485	0.42 (0.10–1.85)	0.2522	1.64 (0.59–4.54)	0.3394

The statistical analysis was performed with the Cox proportional hazards regression model to indicate significance (at *p* < 0.05 level).

aHRs: adjusted hazard ratios; cHRs: crude hazard ratios; CI: confidence interval; AI^: alcoholic intoxication; HCPS*: history of cardiac or pulmonary surgery; SS&: Sjögren’s syndrome; other abbreviations are as described in Table 1.

**Table 4:** Prediction of occurrence of atrial fibrillation.

	cHRs(95% C.I.)	*p*-value	aHRs(95% C.I.)	*p*-value
Gout vs. matched	1.57 (1.47–1.69)	<0.0001	1.38 (1.27–1.48)	<0.0001
Age	1.27 (1.07–1.51)	0.0052	1.24 (1.04–1.48)	0.0195
Comorbidities				
Diabetes Mellitus	1.49 (1.30–1.71)	<0.0001	1.15 (0.99–1.33)	0.0694
CKD	2.30 (1.61–3.29)	<0.0001	1.44 (0.98–2.10)	0.0619
HTN	1.79 (1.62–1.99)	<0.0001	1.24 (1.10–1.39)	0.0004
COPD	1.35 (1.11–1.64)	0.0025	0.98 (0.79–1.21)	0.8308
CAD	2.13 (1.89–2.42)	<0.0001	1.52 (1.32–1.75)	<0.0001
History of cardiac or pulmonary surgery	6.00 (1.34–26.79)	0.0190	3.09 (0.65–14.65)	0.1563
Alcoholic intoxication	2.00 (0.60–6.64)	0.2577	1.62 (0.47–5.65)	0.4487
Sleep apnea	1.40 (0.44–4.41)	0.5655	1.01 (0.29–3.53)	0.9906
CHF	3.80 (3.06–4.71)	<0.0001	2.75 (2.19–3.45)	<0.0001
Rheumatoid arthritis	1.52 (0.95–2.43)	0.0813	1.06 (0.64–1.75)	0.8304
SLE	2.99 (0.61–14.82)	0.1791	2.29 (0.40–12.97)	0.3508
Scleroderma	—	—	0.48 (0.03–9.49)	0.6316
Sjögren’s syndrome	—	—	1.17 (0.49–2.78)	0.7251

The statistical analysis was performed with the Cox proportional hazards regression model to indicate significance (at *p* < 0.05 level).

aHRs: adjusted hazard ratios; cHRs: crude hazard ratios; CI: confidence interval; other abbreviations as in Table 1.
